# Shear-wave elasticity measurements of three-dimensional cell cultures for mechanobiology

**DOI:** 10.1242/jcs.186320

**Published:** 2017-01-01

**Authors:** Po-Ling Kuo, Ching-Che Charng, Po-Chen Wu, Pai-Chi Li

**Affiliations:** 1 Graduate Institute of Biomedical Electronics and Bioinformatics, National Taiwan University, Taipei 10617, Taiwan; 2 Department of Electrical Engineering, National Taiwan University, Taipei 10617, Taiwan; 3 Department of Rehabilitation, National Taiwan University Hospital, Taipei 10002, Taiwan

**Keywords:** Matrix elasticity, Matrix remodeling, Three-dimensional

## Abstract

Studying mechanobiology in three-dimensional (3D) cell cultures better recapitulates cell behaviors in response to various types of mechanical stimuli *in vivo*. Stiffening of the extracellular matrix resulting from cell remodeling potentiates many pathological conditions, including advanced cancers. However, an effective tool for measuring the spatiotemporal changes in elastic properties of such 3D cell cultures without directly contacting the samples has not been reported previously. We describe an ultrasonic shear-wave-based platform for quantitatively evaluating the spatiotemporal dynamics of the elasticity of a matrix remodeled by cells cultured in 3D environments. We used this approach to measure the elasticity changes of 3D matrices grown with highly invasive lung cancer cells and cardiac myoblasts, and to delineate the principal mechanism underlying the stiffening of matrices remodeled by these cells. The described approach can be a useful tool in fields investigating and manipulating the mechanotransduction of cells in 3D contexts, and also has potential as a drug-screening platform.

## Abstract (precis)

**Summary:** Use of a non-direct-contact platform for measurement of the spatiotemporal dynamics of matrix elasticity when remodeled by cells cultured in three-dimensional contexts.

## INTRODUCTION

A common feature shared by tissues in physiological and pathological conditions is that the composition and organization of the extracellular matrix (ECM) – a three-dimensional (3D) complex network supporting the tissues – are dynamically remodeled by both invading and resident cells in response to various stimuli. This produces temporal and spatial variations in the ECM elasticity, which in turn potentiate the pathophysiological conditions. For example, tissues undergoing maturation, repairing, aging and malignant transformation are generally characterized by ECM stiffening, which also promotes these processes (Butcher et al., 2009; Calvo et al., 2013; Collinsworth et al., 2002; Jacob, 2003; Kass et al., 2007; Klein et al., 2009). Mechanobiology delineates the mechanotransduction governing such cell-driven, matrix-coupled remodeling processes, which are best studied using *in vivo* or 3D *in vitro* models (Cox and Erler, 2011); the latter offers several advantages over the former, including lower cost and variability, fewer ethics issues, ease of standardization, and tunable ECM conditions using state-of-the-art technologies (Butcher et al., 2009; Ringe and Sittinger, 2009). Methods that allow quantitative monitoring of the temporal and spatial dynamics of the elasticity of 3D matrices are thus indispensable to providing a mechanistic understanding of cell–matrix interactions in the context of various normal and abnormal states.

The elasticity of an ECM is typically measured by applying a force to a portion of it and measuring the resulting deformation. Standard techniques include atomic force microscopy, nanoindentation and shear rheology. The first two methods probe the elasticity of the material surface with superb spatial resolution, but cannot measure elasticity variations inside the material. Shear rheology involves applying periodic torsions to a sample in order to interrogate the matrix viscoelasticity at various strain rates. However, these measurements are limited to a bulk scale, and the sample is held between torsion plates throughout the experiment, which precludes sample swapping and long-term cell culture. Furthermore, all of these techniques need to directly contact samples during measurements, which can significantly affect the measured values if the experiments are not carefully designed (Cox and Erler, 2011).

Here, we report an *in vitro* platform combining 3D cell culturing and ultrasound-based shear-wave elasticity imaging (SWEI) to evaluate the dynamics of the elasticity of an ECM remodeled by cultured cells. SWEI is a recently developed technique that allows noninvasive and long-term monitoring of changes in tissue stiffness (Gennisson et al., 2010; Sarvazyan et al., 1998). The main concept of SWEI is to quantify the elasticity of a sample based on the speed of a shear wave propagating in it. Stiffer samples are more resistant to deformation and hence are characterized by faster shear waves. Shear-wave velocity has thus been adopted as a surrogate for tissue elasticity in many commercial devices because it is directly related to the shear modulus of elasticity (Nenadic et al., 2011). The ultrasound-based measurements also allow spatially differentiating elasticity changes in heterogeneous samples, particularly over the sample thickness. However, the application of SWEI to *in vitro* 3D models has not been addressed. A main obstacle of applying SWEI to *in vitro* studies is the frequency of the ultrasound beams that generate shear waves. The typical frequency of a clinical ultrasound imaging system is 2–10 MHz, which is ideal for imaging tissues at a scale of centimeters but inappropriate for 3D cell culture systems which have a scale of a few millimeters (Griffith et al., 2005). We overcome this by using a high-frequency imaging transducer (40 MHz), which provides spatial resolution below 100 μm. Moreover, a high-frequency transducer (20 MHz) with a small focal spot size is employed to generate shear waves in the constructs. As the focal spot size decreases, the characteristic frequencies of the induced vibration increase. Thus, at a given shear-wave speed, the mechanical properties of the constructs were probed by waves of smaller wavelengths, which enhances spatial differentiation of elasticity distribution in inhomogeneous constructs.

## RESULTS

### Characteristics of propagating elastic waves

**Fig. 1.**
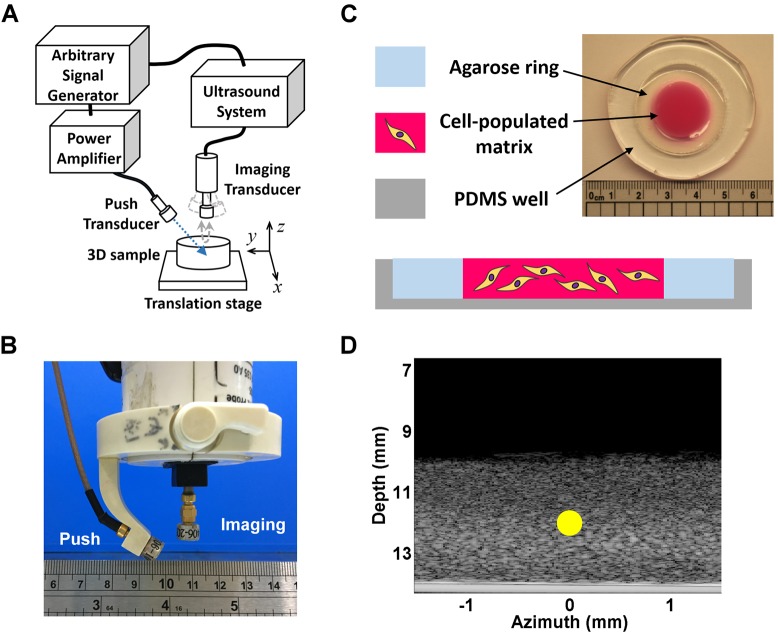
**Schematics and images of the platform.** (A) Schematic of the SWEI setup. (B) Setup of the push and imaging transducers. The transducers were interconnected with a plastic jig to maintain the transducer heads on the surface of a sphere of radius 12 mm to ensure the two transducers were in a confocal condition. (C) Schematic and photograph of the 3D culture system. (D) B-mode image of a hydrogel for 3D cell culture, highlighting the focus of the push transducer (yellow circle).

**Fig. 2.**
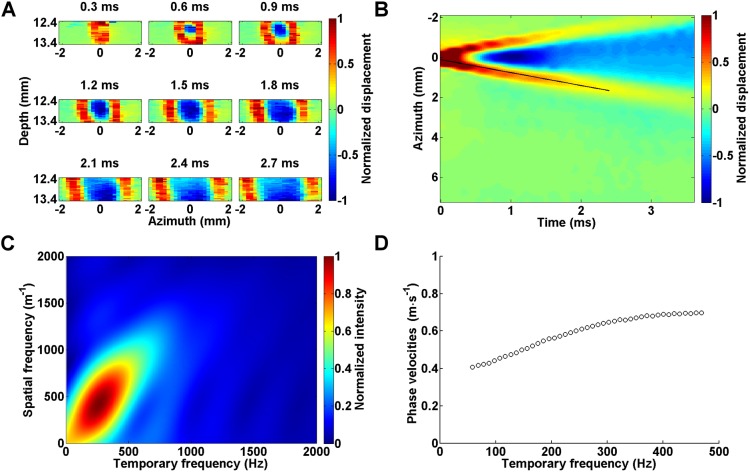
**Spatiotemporal information from elastic waves propagating in a 2 mg ml^−1^ collagen matrix cultured with CL1-5 cells.** (A) Amplitude renderings of the elastic-wave-induced axial displacements as a function of azimuth positions sampled at 9 time points. Wave peaks and valleys are depicted by red and blue color, respectively, and are normalized to their maximums. As time passed, the elastic waves propagated away from the focal path of the push transducer, which was set as the origin of the azimuth axis. (B) Spatiotemporal map of matrix displacement as the elastic wave propagated, with the group velocity estimated by a least-square linear fit to the wave peaks, indicated by the black line. (C) K-space representation of the axial displacements normalized to the maximum as a function of spatial and temporal frequency. (D) Dispersion curve of phase velocities as a function of temporal frequency.

Fig. 1A depicts a schematic of the custom-made, high-frequency SWEI system whose main components are a 20 MHz single-element ultrasonic transducer (used as the push transducer) and a 40 MHz single-element transducer (used as the imaging transducer) that both had a depth of field of 1.47 mm and the same focal depth of 12 mm. The two transducers were carefully aligned using a custom-made, plastic-holding frame such that sound waves delivered from them were focused on the same plane, as shown in Fig. 1B. Fig. 1C depicts the schematic and photograph of the 3D cell culturing system, which was mainly composed of collagen. Biocompatible sound-scattering material, such as silica, was added to the gel to improve the backscattering signals when the sample was scanned with ultrasound. The cell–collagen matrix was fabricated as a 3–4-mm-thick disc with a diameter of 20 mm inside a polydimethlysiloxane (PDMS) well with the gel bottom covalently bonded to the well surface. A 3% agarose ring was fabricated between the side walls of the cell–collagen matrix and the PDMS well to provide a mechanical support of the matrix and facilitate mass transport at its deeper regions. Fig. 1D shows a typical 2D image of the 3D cell culturing system utilizing acoustic backscattering intensity as the image contrast, which is also known as the brightness mode (B-mode) – this mode is commonly used in clinical ultrasound for imaging anatomy of tissues and organs. Focused ultrasound pulses were delivered to the targeted matrix by the push transducer, as indicated by the yellow circle. The acoustic radiation force vibrated the matrix, producing transient elastic waves propagating away from the focus. To detect the vibrational motion perpendicular to the direction of wave propagation (i.e. shear or transverse mode), the temporal profiles of the matrix deformation at the depth direction (i.e. *z*-axis) were mechanically scanned along the azimuth direction (i.e. *x*-axis) by the imaging transducer. Organizing the material motions acquired at various azimuth positions with respect to the time since generation of the elastic wave yielded a time series of the displacement data, which appeared as plane waves when the matrix was generally isotropic (Fig. 2A). Combining the time series of the deformation data yielded a spatiotemporal map of the deformation profiles around the region of interest (ROI) (Fig. 2B). For a generally isotropic material as shown in Fig. 2A, the traveling wave crests exhibit a linear trend on the spatiotemporal map, and the velocity of the propagating amplitude envelop (i.e. the group velocity) could then be approximated by the slope of the wave crests, as indicated by the black line in Fig. 2B.

Given that the 3D cell-populated hydrogel consists of both solid and fluid phases, its mechanical properties have a viscoelastic nature and depend on the frequency of deformation, as determined by the elastic wave frequency. To analyze the changes in the wave speeds with respect to various frequencies (i.e. dispersion), we applied two-dimensional (2D) Fourier transform to the spatiotemporal map shown in Fig. 2B as previously described (Bernal et al., 2013). Fig. 2C depicts the transformed results as a function of various temporal and spatial frequencies (i.e. wavenumber or wavelength^−1^). The intensity at individual pixels represents the relative contribution of the wave component at the corresponding wavenumber and temporal frequency to the propagating wave. Thus, the main wavenumber at a selected temporal frequency was found by identifying the intensity maximum at that frequency. Dividing the temporal frequency by the wavenumber corresponding to the intensity maximum yielded the phase velocity of the propagating wave at various temporal frequencies, as shown in Fig. 2D. Note that the lowest frequency of the phase velocity is constrained by the spatial range of wave measurement and the wavelength (Bernal et al., 2013). For example, given a phase velocity of 0.4 m s^−1^, the wavelength is on the order of 8 mm at 50 Hz, and the wave dynamics cannot be properly detected if the measured field along the azimuth direction is smaller than 8 mm (i.e. only waves that oscillate at frequencies higher than 50 Hz can be determined). Also note that the matrix deformations were nearly negligible at temporal frequencies higher than 500 Hz when a 3 dB threshold was applied to the transformed data. The average of the phase velocities shown in Fig. 2D was 0.68 m s^−1^, which is very close to that determined by the group velocity as shown in Fig. 2B (0.66 m s^−1^).

**Fig. 3.**
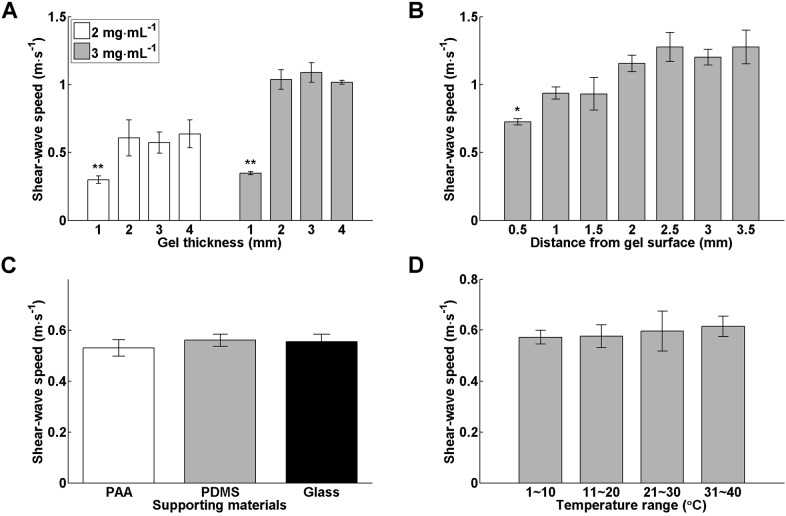
**Changes in wave speeds in various conditions.** (A) Group velocities of the elastic waves propagating in 1–4-mm-thick collagen matrices of different concentrations (*n*=3 in individual conditions). (B) Variations of group velocities measured in 4-mm-thick 3 mg ml^−1^ collagen matrices (*n*=4) at various focal positions. (C) Group velocities measured in 4-mm-thick 2 mg ml^−1^ collagen matrices that were bonded to substrates of various rigidities (*n*=4 for individual conditions). (D) Group velocities measured in 4-mm-thick 2 mg ml^−1^ collagen matrices (*n*=3) at various temperatures. Data in all panels are presented as mean±s.d. *P<0.05; **P<0.01 [one-way ANOVA (A) and Tukey's *t*-test (B)]. For C,D, there was no significant difference between the wave speeds across various conditions.

By characterizing the dispersion of phase velocity, we further confirmed that the elastic waves propagating in our 3D matrices were shear waves. When elastic waves propagate in a thin disc that has a thickness close to or smaller than the wavelength of the propagating waves, the waves might be guided along the disc due to interference and coupling of the waves reflected from the disc boundaries. The resulting superimposed waves form Lamb waves, which oscillate particles in an elliptical path as the wave is guided along the disc, such that the particles at the upper and bottom part of the disc rotate in the opposite direction (i.e. clockwise versus counterclockwise) in the symmetric mode, and that the particles at the two parts rotate in the same direction in the antisymmetric mode. Consequently, the disc deformation over its thickness is characterized by a periodic expansion and compression, and upward and downward undulation in the symmetric and antisymmetric mode, respectively. It is important to distinguish whether the propagating elastic waves are shear or Lamb waves because the relationships between the wave speed and material elasticity are different in both conditions (Royer and Dieulesaint, 1996). Lamb waves typically occur in discs when the disc boundaries are not covalently bonded to a solid surface, which is different from our conditions. Furthermore, Lamb waves are highly dispersive and the phase velocities of the guided waves are usually dispersed to higher temporal frequency regimes, typically around 500–2000 Hz (Brum et al., 2012; Nguyen et al., 2012, 2011; Urban et al., 2013), which again is not seen in our cases (Fig. 2C). Another important feature of Lamb waves is that the phase velocity is proportional to the square root of structure thickness (Royer and Dieulesaint, 1996). To examine whether the wave speeds measured in our matrices depended on the matrix thickness, we conducted a series of wave speed measurement using collagen matrices of various concentration and thickness. Given that the average of the phase velocities differed negligibly from the group velocity, we used the latter as an estimate of the speed of the propagating waves. Fig. 3A shows that at the same collagen concentration, the group velocity changed negligibly as the gel thickness increased from 2 to 4 mm. However, the wave speeds dropped when the gel thickness decreased to 1 mm. This might be due to the fact that the depth of field of the ‘push beam’ was about 1.5 mm, which would be expected to elicit slow-propagating surface waves when the gel thickness was smaller than the focal size. Taken together, these data indicate that the elastic waves propagating in our disc-shaped matrices were purely shear waves.

### Effects of repeated measurements, focal positions, hydrogel boundaries and temperatures on wave speed measurement

We first examined the reproducibility of the shear-wave data measured at the same position in the 3D matrices. The shear-wave speeds were repeatedly measured five times at two randomly chosen positions in a 3 mg ml^−1^, 3-mm-thick collagen matrix. The group velocities of the induced shear waves were 1.11±0.05 m s^−1^ and 1.10±0.05 m s^−1^ (mean±s.d.; *n*=5) for the two positions, respectively. The small variations in the mean values of shear-wave speeds between the two positions (<1%) and the small ratios of the standard deviation to the mean values (<5%) convinced us that the gel was homogenous and the data were reproducible. We then asked whether, given an isotropic matrix such as a collagen gel, changes in the focal position of the push beam, the mechanical properties of surface attached to the gel bottom and the temperature during measurement could affect the speed of the induced shear waves. Fig. 3B shows the group velocities of shear waves induced by acoustic radiation forces delivered in 4-mm-thick 3 mg ml^−1^ collagen matrices at focal positions varying from 0.5 to 3.5 mm away from the top surface of the matrices (i.e. the water–gel interface). Statistical analysis revealed that the speeds of shear waves induced at a focus 0.5 mm apart from the water-gel interface were significantly slower than those induced at deeper positions. This was to be expected because setting the focus at positions close to the water–gel interface might elicit surface acoustic waves that propagate much slower than shear waves. By contrast, the shear-wave speeds changed insignificantly as the waves were induced at deeper positions. To investigate whether the mechanical properties of the hydrogel boundaries affects the shear-wave speeds, we modified the PDMS surface to which the bottom of the hydrogel was bonded using materials of varied rigidities. Fig. 3C shows the group velocities of shear wave propagating in 2 mg ml^−1^, 3–4-mm-thick collagen matrices that were attached to silanized glass coverslips (the hardest surface), PDMS (medium hardness) and polyacrylamide gels (the softest surface), respectively. Statistical analysis indicates that there were no significant changes in the shear-wave speeds measured in the collagen gels constrained by boundaries of varied rigidities. Given that the shear-wave measurements were conducted in a culture hood at room temperature, we also wondered whether the shear-wave speeds were affected by changes in environmental temperature. Fig. 3D demonstrates the group velocities of shear wave propagating in 2 mg ml^−1^, 4-mm-thick collagen matrices that were maintained at a constant temperature ranging from 0 to 40°C during the measurement using a dry cooler and a thermometer for temperature monitoring. The shear-wave speeds did not vary significantly across the applied temperatures. Collectively, these data indicate the shear-wave measurement using our system was robust and relatively insensitive to changes in the environmental factors, such as temperature and boundary conditions.

### Data correlation with shear rheology

**Fig. 4.**
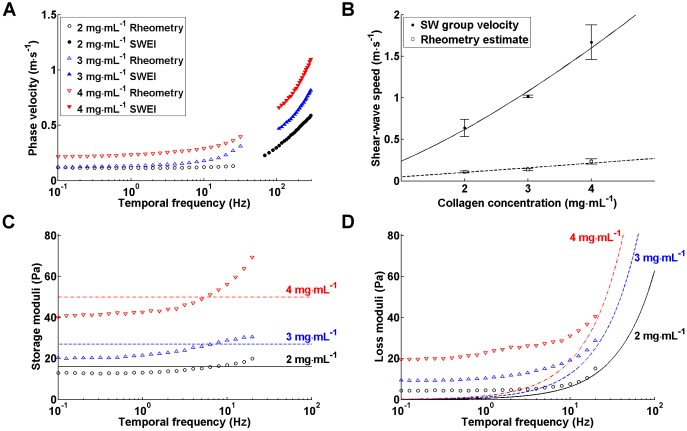
**Data compatibility between SWEI and shear rheology.** (A) Dispersion curves describing phase velocities of collagen matrices of concentration 2–4 mg ml^−1^ that were derived from shear rheology and SWEI. Data points represent the mean values from three independent measurements; the standard deviations were not drawn to improve image clarity. The phase velocities of shear wave at low-frequency regime are generally not measurable, and the lowest frequency of the measurable velocities increases as the materials become stiffer. (B) Collagen concentration dependence of the group velocities measured from SWEI and the phase velocities derived from the rheometry data at 1 Hz. The shear-wave measurements were conducted in 4-mm-thick collagen matrices of various concentrations. Data are presented as mean±s.d. and the sample number was three in each condition. The solid and dash line represent the fitting function *v*_s_=0.24*c*^1.37^ and *v*_s_=0.05*c*^1.05^, respectively. (C) Experimental and model-fitted results of the storage modulus (*G*′) obtained by the rheological and SWEI approaches, as indicated by the key in A. The Voigt-fitted storage moduli are 16, 27 and 50 Pa for the 2, 3 and 4 mg ml^−1^ collagen matrices, respectively. (D) Experimental and model-fitted results of loss modulus (*G*″) obtained by the rheological and SWEI approaches, as indicated by the key in A. The Voigt-fitted viscosity constants are about 0.1, 0.2 and 0.3 Pa·s for the 2, 3 and 4 mg ml^−1^ collagen matrices, respectively.

To determine whether the elastic properties of the 3D matrices measured by the SWEI system were quantitatively reasonable, we compared the SWEI results with those derived from shear rheology, which is commonly referred to as the standard technique for viscoelasticity measurement of biomaterials. Shear-wave and rheological measurements were conducted in collagen matrices of concentration varying from 2 to 4 mg ml^−1^. To facilitate data comparison, the phase velocities derived from both techniques were plotted against temporal frequency ranging from 0.1 to 300 Hz (Fig. 4A). Given that slippage between the rotating plate of the rheometer and the sample usually occurs at higher oscillating frequencies, and that it is difficult to obtain the phase velocities of shear waves at a low-frequency regime, there was inevitably a frequency gap in the curve generated by both techniques. Nevertheless, both data exhibit frequency-dependent changes as the collagen concentration increased. Fig. 4B shows the quantitative relations between the wave speeds *v*_*s*_ and collagen concentrations *c* using power fits. The group velocity derived from SWEI increased in proportion to *c*^1.37^, whereas the phase velocity estimated from the rheological measurement at 1 Hz – the typical frequency that cells probe surrounding matrix (Engler et al., 2008) – was proportional to *c*^1.05^. Note that for a purely elastic, isotropic material, the shear modulus *μ* is readily determined as 
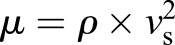
, where *ρ* represents the mass density. Hence, the shear modulus of the collagen gel is proportional to *c*^2.74^ in the SWEI measurement, provided that the collagen matrix is isotropic. This concentration dependence is very close to that reported by a recent study (*μ*∝*c*^2.7^) (Stein et al., 2011), but somewhat different from that of another obtained using shear rheology (*μ*∝*c*^2.1^) (Yang et al., 2009). In contrast, the power law scaling for the concentration dependence in the rheometry data was very close to that reported by Yang et al. in 2009, when the shear modulus was either assigned by the storage modulus *G*′ (*μ*∝*c*^2^), or estimated from the phase velocity using the isotropic assumption (*μ*∝*c*^2.1^). The 5–10-fold disparity between the SWEI and rheometry data might result from the difference of applied frequencies, given that the phase velocity increases as the frequency increases (Fig. 4A).

To further correlate the SWEI data with the rheometry data, we fitted the SWEI data with the Kelvin–Voigt model, and compared the viscoelastic parameters derived from the fitted model with those measured by rheometry. The Kelvin–Voigt model approximates the material viscoelasticity as a spring with an elastic constant *μ*_V_ connected in parallel with a dashpot that has a viscous constant *η*_V_. This model is widely used for biomaterials and its main advantage is that the fitted shear elasticity *μ*_V_ and viscosity *η*_V_*ω* directly correspond to the storage moduli *G*^′^(*ω*) and loss modulus *G*(*ω*) measured by rheometry, respectively, where *ω* represents the angular frequency. The results show that the fitted storage moduli were within the range of experimental data (Fig. 4C), but there is greater disparity between the values of fitted and measured loss moduli at lower frequencies (Fig. 4D). This was to be expected because the Voigt loss modulus drops linearly with decreasing frequency. Nevertheless, these results suggest that the SWEI is a compatible alternative to shear rheology in elasticity measurements of common biological materials.

### Characterization of anisotropic materials

**Fig. 5.**
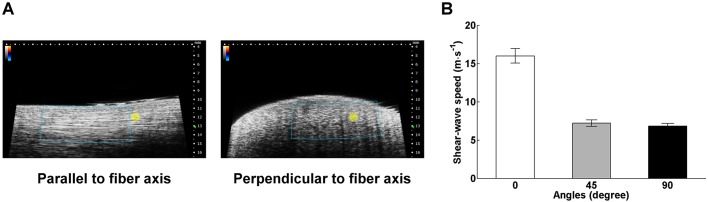
**Images and shear-wave measurements of an anisotropic material.** (A) B-mode images of a bovine tendon acquired at direction in parallel and perpendicular to the longitudinal axis of the sample. The image acquired along the longitudinal direction is characterized by parallelly aligned, high echogenicity lines, whereas the perpendicular image is not. The blue box and the yellow circle in both images represent the ROI of shear-wave measurements and the focus of the push transducer, respectively. (B) The shear-wave speeds measured at directions with an inclined angle between the scanned plane and the longitudinal axis of the sample at 0, 45, and 90 degrees, respectively. Data are presented as mean±s.d. and averaged from 5 measurements in each direction.

We also demonstrated that the presented system is capable of differentiating changes in elasticity at various orientations in biomaterials of anisotropic nature. Here, we took a bovine tendon as an example because it consists of collagen fibers that are well arranged in parallel. Fig. 5 exhibits the fiber-orientation dependence of shear-wave speeds in the tendon. The shear-wave speeds were 16.0±0.9, 7.2±0.4, and 6.8±0.3 m s^−1^ (mean±s.d.; *n*=5) when measured along directions with an inclined angle with the fiber orientation at 0°, 45° and 90°, respectively. Statistical analysis using one-way ANOVA revealed that there is a significant difference in the mean wave speeds measured along various directions (*P*<10^−10^). Note that tendons are typically modeled as transversely isotropic materials and the derivation of the elastic properties along various fiber orientations requires additional data such as the wave speeds of longitudinal mode (Kuo et al., 2001). Nevertheless, our data suggest that shear-wave speed can be a reasonable surrogate for the elastic characteristics of anisotropic materials.

### Spatiotemporal dynamics of matrix elasticity

**Fig. 6.**
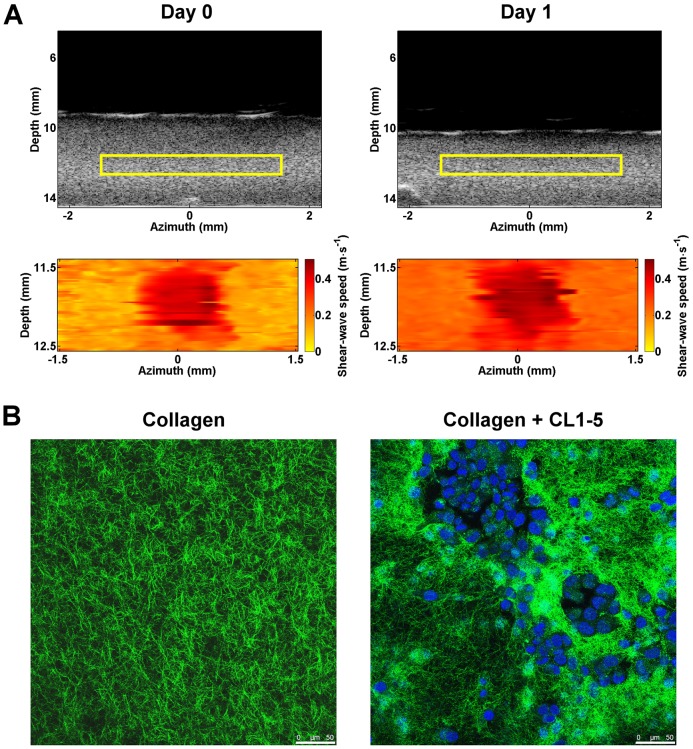
**ECM remodeled by advanced lung cancer cells.** (A) B-mode images and spatial maps of shear-wave speeds acquired from a 4-mm-thick 1 mg ml^−1^ collagen matrix cultured with CL1-5 lung cancer cells at the time immediately (day 0) and 24 h (day 1) after gelation. The areas corresponding to the speed maps are highlighted by the yellow rectangles in the B-mode images. (B) Confocal images of the cell-populated matrix and a pure 1 mg ml^−1^ collagen matrix. Green, collagen; blue, nuclei. Scale bars: 50 µm.

An attracting advantage of SWEI is its ability to provide the spatiotemporal changes in the elastic properties of cell-populated matrices. This information is important to shed light on fields involving the mechanical interactions between cells and the surrounding matrix, such as tissue engineering, tissue development, wound healing and tumorigenesis. Fig. 6A shows an example of the spatiotemporal dynamics of the shear-wave speeds measured from a 1 mg ml^−1^ collagen matrix remodeled by the human lung adenocarcinoma cells CL1-5, which are highly invasive, mesenchymal-transformed cancer cells (Chu et al., 1997; Liou et al., 2014; Shih and Yang, 2011). The cancer cells were seeded into the matrix at a density of 10^6^ cells per ml. The PDMS well encompassing the cell-populated matrix was marked with a line transecting the matrix center and shear-wave measurements were conducted by imaging the matrix along a cross-sectioned plane containing the line on the same day of cell seeding (day 0) and 24 h after seeding (day 1). As shown in the corresponding B-mode images, the cell-populated matrix contracted and became thinner as the culture day increased. Given that the bottom of the cell-populated matrix was immobilized from the PDMS well, calculating the difference in gel thickness between B-mode images acquired at different culture days yielded an estimate of the percentage of matrix contraction, which was ∼20% after culturing the cells for 1 day. In line with this, is the finding that the shear-wave speeds of the cell-populated matrix increased as the culture day increased. Note that the high-amplitude area at the speed map center correspond to the focus of the push beam, which generated forced vibrations of similar magnitude across individual measurements. Given that the shear-wave speeds generally exhibited a homogenous distribution within the ROI, it is reasonable to assume that the cell-populated matrix was elastically isotropic. Assuming the cell-populated collagen matrix as an isotropic, linear elastic material and its mass density as 1060 kg m^−3^, the shear moduli derived from the spatial average of the shear-wave-speed map were 42 and 95 Pa for the matrix measured on day 0 and 1, respectively. Confocal images matching the orientation of B-mode images revealed that the cancer cells formed scattered clusters and reorganized the fibrils into a more condensed network, as compared with those without cells (Fig. 6B). The changes in matrix elasticity and morphology are similar to those of matrix remodeled by cancer-associated fibroblasts (CAF) isolated from advanced breast tumors (Calvo et al., 2013).

### Molecular mechanisms governing changes in matrix elasticity driven by lung cancer cells

**Fig. 7.**
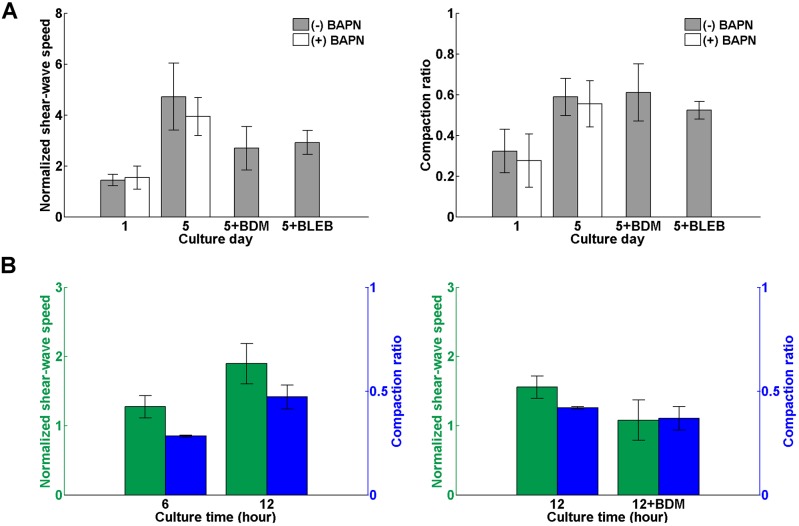
**Changes in elasticity of cell-populated matrices after drug treatments.** (A) Temporal variations in shear-wave group velocities and gel volume compaction measured in CL1-5-populated collagen matrices. The samples were cultured without treatment [(−) BAPN; *n*=10] for 5 days or were treated daily with 350 µM aminopropionitrile [(+) BAPN; *n*=6]. After 5 days of culture, the samples in the normal culture group were either treated with 40 mM BDM for 9 h [5+BDM; *n*=4] or 30 µM blebbistatin [5+BLEB; *n*=5] to hinder cell contraction. (B) Temporal variations in shear-wave group velocities and gel volume compaction measured in collagen matrices cultured with H9C2 cells for 12 h (*n*=4) and those measured in H9C2-populated collagen matrices that were treated with 40 mM BDM for 9 h after culture of 12 h (*n*=3). To facilitate data comparison, the wave speeds and gel thickness measured at various time points in A and B were normalized to those measured immediately after the matrices gelation. Data shown in all figures is presented as mean±s.d.

A potential application of the described platform is to quantitatively address mechanisms underlying the mechanical remodeling of the ECM by advanced cancer cells as shown in Fig. 7. One mechanism that could account for the ECM stiffening is condensation of the collagen network due to volume contraction because collagen gel becomes stiffer as the collagen concentration increases (Motte and Kaufman, 2013; Yang et al., 2009). Other mechanisms that might result in ECM stiffening include cross-linking of the collagen network and stress-induced stiffening of the cell–matrix composite driven by cell traction (Fernandez and Ott, 2008; Lam et al., 2011). Advanced cancer cells such as CL1-5 cells can secrete lysyl oxidase (LOX), an extracellular copper enzyme that oxidizes lysine residues in elastin and collagen to form covalent cross-linking between these fibrous proteins that can stiffen the matrix (Baker et al., 2013; Chou et al., 2013; Erler et al., 2006). It is also well known that cells adapt to environmental stiffness by contracting the actomyosin system, which stiffens the cells and increases the tension in the fibrillar network of the ECM (Crow et al., 2012; Mitrossilis et al., 2010).

To determine the extent to which ECM stiffening is attributable to these mechanisms, we cultured CL1-5 cells with a seeding amount of 1.5×10^6^ cells in 1 mg ml^−1^ collagen matrices for 5 days in two different conditions. The cell-populated matrices in the first condition were treated daily with 350 µM β-aminopropionitrile (BAPN), an irreversible inhibitor of LOX; the matrices in the second condition were cultured as usual but incubated with 40 mM 2,3-butanedione monoxime (BDM), a reversible myosin II ATPase inhibitor that impedes actomyosin contraction, for 9 h, or 30 µM blebbistatin (BLEB), an inhibitor of myosin II that impedes cell traction, for 1 h, at the end of culture. SWEI measurements were acquired at day 0, 1 and 5 after cell seeding. To facilitate data comparison, the group velocities of shear waves were measured at more than five spots for each sample as an individual measurement, spatially averaged and normalized to that derived on day 0. The thickness of the cell-populated matrices measured on different days was also normalized to that obtained on day 0 to calculate the amount of gel compaction. As shown in Fig. 7A, the normalized shear-wave speeds and the volume compaction ratio of the CL1-5-populated matrices increased steadily as the culture days increased. At the end of culture, the normalized shear-wave speeds increased to 4.7±1.3 and 3.9±0.7 (mean±s.d.) for the conditions with normal culture prior to BDM or BLEB treatment (*n*=10) and BAPN treatment (*n*=6), respectively. The normalized speeds dropped to 2.7±0.8 and 2.9±0.5 after BDM and BLEB treatment, respectively. Paired comparison of the speed data prior to and after BDM or BLEB treatment revealed that cell tractions were estimated to account for the ∼40% of the matrix stiffening, whereas ∼20% was attributable to the fibril cross-linking mechanism by comparing the data in normal culture and BAPN treatment conditions. Note that the gel volumes were compacted more than 50% after 5 days of culture. Application of BDM or BLEB loosened the gel but did not immediately bring the gel volume back to that measured at earlier days. Assuming that the synthesis and degradation of collagen were negligible in the cell-populated collagen gel, a 50% compaction of the gel volume would simply double the collagen concentration to 2 mg ml^−1^. The averaged group velocity of shear waves propagating in 2 mg ml^−1^ collagen matrices was about 0.5–0.6 m s^−1^, which was about the same as that measured in the BDM-treated matrices (0.56±0.03 m s^−1^) and BLEB-treated matrices (0.55±0.06 m s^−1^). This suggests that the BDM and BLEB treatment had similar effects on gel relaxation as well as that the remaining 40% of the ECM stiffening is probably attributable to condensation of the collagen network.

### Molecular mechanisms governing changes in matrix elasticity driven by cardiac myoblasts

Another attractive application of the described platform is to evaluate the maturation of engineered cardiac muscle tissues by quantitatively monitoring the elasticity changes in the engineered tissues. The maturation of developing cardiac muscle cells is known to be closely regulated by tissue elasticity. The elastic modulus of embryonic heart tissue is about 11–12 kPa (Engler et al., 2008; Jacot et al., 2010), and is stiffened to 18–39 kPa at maturation (Berry et al., 2006; Jacot et al., 2010). When cultured on a substrate with a elasticity mimicking that of developing myocardium, maturing cardiac muscle cells beat faster and longer and have the best chance to form striated myofibrils (Bajaj et al., 2010; Engler et al., 2008; Jacot et al., 2008). Developing cardiac muscle cells probably probe environmental elasticity by generating traction on adhering substrate. However, it has not been clearly shown how the developing cardiac muscle cells modify the elasticity of their environment.

To demonstrate the feasibility of using our platform to investigate the mutual interaction between developing cardiac muscle cells and their environment, we cultured cardiac myoblasts H9C2 with a seeding amount of 7.5×10^5^ cells in ∼4-mm-thick 2 mg ml^−1^ collagen matrices and monitored the elasticity changes in the cell-populated matrices. Fig. 7B shows the temporal variations in the spatially averaged shear-wave speeds and matrix volume normalized to that measured at the beginning of culture. It was seen that H9C2 cells substantially contracted and stiffened the matrix after 12 h of culture. The rapid contraction of the H9C2-populated collagen matrices suggests that the matrix stiffening might be primarily attributed to cell traction. We conducted another series of experiments and found that blocking actomyosin contraction of the H9C2 cells using BDM after 12 h cell culture substantially reduced the matrix elasticity, which was the same as that measured at the beginning of culture. Again, the matrix thickness did not substantially relax after the BDM treatment, which suggests that the observed changes in matrix elasticity was predominantly attributable to the stress-induced stiffening resulting from the actomyosin contraction of the H9C2 cells, rather than the condensation of the collagen network. Given that matrix stiffening further enhances muscle cell maturation, these data indicate that a feed-forward, self-reinforcing loop exists between the cardiac myoblasts and their environment, which promotes the myoblast maturation.

## DISCUSSION

In the present work, we describe a platform that adopted a ultrasonic-shear-wave-based system for elasticity imaging of 3D cell culture. The described platform is useful in providing the spatiotemporal dynamics of elasticity in cell-populated matrices and appears to be a promising tool for mechanistic studies regarding the mechanotransduction between cells and the environment in 3D contexts. We also demonstrated that the elasticity measurements using our platform for collagen — a very commonly used biomaterial — were quantitatively correlated with those derived from shear rheology. The fabrication process of the 3D culturing system coupled with the imaging platform is simple and straightforward. The high-frequency transducers enhance the spatial resolution of elasticity measurements of the 3D samples. Given the current setup, the spatial resolutions of the shear-wave speed map are ∼190 and ∼19 µm along the azimuth and depth direction, respectively, and the measurable shear-wave speeds range from 0.03 to 30 m s^−1^, which corresponds to isotropic hydrogels of shear moduli of ∼1 Pa to hundreds of kPa and would be expected to be applicable for most hydrogels utilized in *in vitro* models.

However, there are several limitations in the proposed approach. The elasticity of the material was derived from the speed of the bulk shear waves induced inside the 3D matrix of thin discoid shape, which is the most commonly used geometry for *in vitro* 3D models. The focal-shape of the push beam that vibrates the matrix appears as an elongated spheroid with a vertical size of about 1.5 mm. Thus, when the vibrations were induced at depths less than 0.75 mm from the sample surface, or the sample thickness was smaller than the focal size, surface acoustic waves propagating at the water–gel interface can be generated. Misidentification of the surface waves as shear waves would underestimate the material elasticity, because the speed of the surface waves is generally slower than that of the shear waves. To avoid errors resulting from the slow-propagating surface waves, we suggest that the thickness of the targeted cell-populated hydrogels should not be less than 2 mm. Nonetheless, we anticipate that this limitation can be alleviated by developing push transducers with a smaller focal size, or adding into our platform the mathematical framework characterizing material elasticity using the speed of surface waves, such as that recently reported in Kazemirad et al. (2013). Another disadvantage of our system is the constraint of obtaining phase velocity at low frequencies due to the limited scale of measuring field. This is because the measuring field in our system is about the size of most *in vitro* models and measuring shear-wave speeds at high frequencies is important to improve the spatial resolution. Nevertheless, this can be improved by extrapolating low-frequency data using a specific theological model, although doing this increases computation complexity.

The platform described herein is readily generalizable to mechanistic studies of cell–ECM dynamics perturbed by the use of various cell lines, primary cells and even coculturing of different types of cells. In the present study, we investigated the mechanisms underlying matrix stiffening driven by highly invasive lung cancer cells and cardiac myoblasts. Exploring ECM changes during tumor progression has been seen as a promising target for drug development because the interplay between cancer cells and stroma play an essential role in tumor growth and metastasis (Cirri and Chiarugi, 2011; Lu et al., 2012; Pietras and Ostman, 2010; Sounni and Noel, 2013). A hallmark of ECM changes associated with advanced cancers is stiffening, which is an essential process to promote cancer progression, migration and metastasis (Cirri and Chiarugi, 2011; Kostic et al., 2009; Liou et al., 2014; Otranto et al., 2012; Pietras and Ostman, 2010). The current consensus is that the stiffening of a cancerous ECM is driven by the contraction of CAFs and pericytes, which are the most abundant stromal cells associated with cancers, as well as the increased deposition and cross-linking of matrix proteins mediated by the fibroblasts (Cirri and Chiarugi, 2011; Koumoutsakos et al., 2013; Otranto et al., 2012; Pietras and Ostman, 2010). However, our data indicate that highly invasive cancer cells alone behave similarly to the CAFs in stiffening and remodeling of surrounding matrix to facilitate their invasion through the matrix.

In summary, an ultrasonic shear-wave-based platform has been developed and validated for its efficacy in quantitatively evaluating the spatiotemporal dynamics of the elasticity of a matrix remodeled by cells cultured in a 3D environment. We anticipate that this approach will benefit several research fields related to the dynamic reciprocity between cells and their environment, such as studies in tissue fibrosis, tumor evolution, wound healing, tissue engineering and stem cell differentiation.

## MATERIALS AND METHODS

### 3D cell culture

A circular PDMS well was fabricated by casting a degassed mixture of PDMS prepolymer (Sylgard 184, Dow Coring) and curing agent at a ratio of 10:1 by weight into a polymethylmethacrylate (PMMA) mold, followed by baking at 65°C for 4 h. The cured PDMS replica had an interior height of 4 mm and diameter of 34 mm. The interior surface of the PDMS well was silanized with a solution of 10% (3-Aminopropyl) trimethoxysilane (APTES) (440140, Sigma-Aldrich) in 95% ethanol at 50°C for 1 h after oxygen plasma treatment using an oxygen plasma cleaner (PDC32G, Harrick Plasma, NY), followed by incubation with a 0.5% glutaraldehyde solution at room temperature for 1 h. After the interior surface of the well was washed extensively with deionized water, a 5-mm-thick PMMA disc with a diameter of 20 mm was placed firmly at the center of the interior bottom of the well, and the space between the PMMA disc and the interior wall of the well was filled with a 3% agarose solution, which formed a ring after complete gelation. The PMMA disc was removed thereafter.

For culturing cells in 2 mg ml^−1^ type I collagen matrices, the cell–collagen mixture was prepared by adding 291 µl of collagen stock solution (354249, BD Biosciences, NJ) to a neutralizing buffer containing 49.7 µl of 10× PBS, 150 µl of 3% (w/v) silica (S5631, Sigma-Aldrich) and 6.7 µl of 1 M NaOH, and carefully mixed with 1002.6 µl of the cell suspension (1.5×10^6^ cells ml^−1^) at 0°C. The number of cells in the suspension was adjusted depending on various experimental conditions. For culturing cells in 1 mg ml^−1^ type I collagen matrices, the amounts of collagen stock solution, the 1 M NaOH solution, 10× PBS, and the cell suspension medium (1.3×10^6^ cells ml^−1^) were changed to 145.5, 3.3, 33.2 and 1167.9 µl, respectively. The cell–collagen mixture was carefully added into the disc-shaped space encompassed by the agarose ring in the PDMS well and allowed to polymerize so as to encapsulate the seeded cells in humidified air at 37°C for 2 h. The cell-populated hydrogel–PDMS composite was placed in a 50-mm culture dish, kept in a conventional incubator (37°C, 5% CO_2_), and supplied with cell culture medium composed of 10% fetal bovine serum (FBS) and 1% antibiotics (ABL02, Caisson Laboratories, Logan, UT) in Dulbecco's modified Eagle's medium (DML-10, Caisson Laboratories). The cell culture medium was changed daily. CL1-5 human lung adenocarcinoma cells were derived from the CL1 clonal cell line as described previously (Chu et al., 1997), and were kindly provided by Pan-Chyr Yang (National Taiwan University College of Medicine, Taipei, Taiwan). H9C2 cells were obtained from the ATCC.

To modify the interface bonded to the bottom boundaries of the collagen gels, the interior bottom of the PDMS well was either attached with an APTES-silanized glass coverslip, or a polyacrylamide (PAA)-gel–glass-coverslip composite, after the well was treated with oxygen plasma. The PAA-gel–glass-coverslip composite was made by fabricating a 1-mm-thick layer of PAA gel on the surface of a 30-mm methacrylated glass coverslip as described previously (Kuo et al., 2012). The coverslips was first immersed in a 0.1 M NaOH solution for 10 min, sonicated, rinsed with deionized water, air dried, covered with 1 ml of solution composed of 20 µl of 3-(trimethoxysilyl)propyl methacrylate (M6514, Sigma-Aldrich, St. Louis, MO, USA), 120 µl of 10% (v/v) acetic acid solution (A6283, Sigma-Aldrich) and 860 µl of 95% (v/v) ethanol for 30 min, washed with ethanol, air dried again and heated to 95°C for 15 min. The PAA gel had final concentrations of 8% (w/v) acrylamide (ACR006, Bioshop, Burlington, ON, Canada), 0.5% (w/v) bis-acrylamide (BIS001, Bioshop) and 5% (w/v) polyethylene glycol (P6667, Sigma-Aldrich), and the gelation was catalyzed by the addition of 0.1% (v/v) ammonium persulfate (A2941, Sigma-Aldrich) and 0.1% (v/v) tetramethylethylenediamine (TEM001, Bioshop). Another glass coverslip silanized with dichlorodimethylsilane (440272, Sigma-Aldrich) was placed on top of the PAA prepolymer solution to ensure that the solution formed a hydrogel with a uniform thickness. After gelation, the dichlorodimethylsilane-treated coverslip was removed, and the surface of the PAA gel was covered with 500 µl of 1-mg/ml sulfo-SANPAH (22589, Thermo Scientific, Waltham, MA) and exposed to UV light (wavelength=365 nm) for 10 min. The sulfo-SANPAH treatment facilitated covalent bonding between the surface of the PAA gel and a subsequently added cell–collagen mixture.

### Shear-wave speed measurement

The SWEI system consisted of an arbitrary-signal generator (AFG 3252C, Tektronix, Beaverton, OR), an ultrasound system (Prospect, S-sharp, New Taipei City, Taiwan), an RF power amplifier (250A250A, Amplifier Research, Souderton, PA) and two custom-made single-element transducers (Acoustic Sensor Corp, New Taipei City, Taiwan) with center frequencies of 20 and 40 MHz. The 20 MHz transducer was used as a push transducer to generate pulsatile shear waves in targeted medium, and the 40 MHz transducer was used to image the wave propagation. The two transducers had the same focal depth of 12 mm, and they were carefully aligned such that sound waves delivered from them were focused on the same plane. The sample for imaging was placed on a two-axis translation stage with resolution of 10 µm. Image acquisition was conducted in a culture hood to ensure an aseptic condition and initiated by sending a 100-µs amplified tone burst to the focus of the push transducer. To detect the displacements along the depth direction (i.e. axial displacements) induced by the delivered shear wave propagating along the azimuth direction, M-mode images of the vibrating matrix at a predetermined position were acquired at a pulse repetition frequency of 2 kHz for 20 ms or 10 kHz for 4 ms, whereas the higher sampling frequency was for detection of shear waves propagating at faster speeds. The shear-wave generation and image acquisition were synchronized by the ultrasound system. The imaging transducer was then moved to the next azimuth position and the synchronized process repeated until M-mode images had been acquired from 50 positions equally separated along a 9.5-mm path of the shear-wave propagation. Note that the push beam was kept at the same focus during image acquisition, and the delivered shear waves were assumed to have similar waveforms. The imaged sample was then advanced along the azimuth direction at an increment of 50–150 µm using the translation stage and the image process was then repeated. The *in situ* spatial peak temporal average intensity (*I*_spta_) and spatial peak pulse average intensity (*I*_sppa_), after correcting for the attenuation of the push beams delivered during image acquisition as estimated by a hydrophone (MHA9-150, Force Technology, Denmark), were 62.8 mW/cm^2^ and 66 W/cm^2^, respectively, which satisfies the safety limits required for diagnostic ultrasound. All data were analyzed using functions provided by MATLAB. The M-mode data were demodulated to obtain the baseband, in-phase and quadrature-phase data of individual A-lines. To improve the signal-to-noise ratio, the A-line data were resampled by consecutively averaging a subset of data of length 0.0385 mm (i.e. the gate size) with the next subset chosen from data separated by a distance of 0.019 mm (i.e. the sliding window) along the depth direction. A one-dimensional autocorrelation algorithm was applied to the resampled A-line data successively acquired at the same position to determine the axial displacements induced by the propagating shear waves. Organizing the axial displacements acquired at various azimuth positions with respect to the time since generating the shear wave yielded a time series of the displacement data. Displaying the displacements measured at a chosen depth as a function of time generated a spatiotemporal map of the axial displacements that fully describes the propagation of shear waves along the azimuth direction. To calculate the shear-wave speed at various azimuth positions, the temporal profiles of the axial displacements at equally separated azimuth positions were extracted from the map, and the time spent when the wave propagated along adjacent positions was determined by applying a cross-correlation algorithm to the extracted data. Note that the temporal profiles of the axial displacements extracted at individual positions were interpolated by a factor of 20 using spline interpolation to improve the robustness in estimation of the traveling time. The group velocity at a given position was then determined by dividing the predetermined separation between adjacent positions by the calculated traveling time. The chosen separations between the adjacent positions were 50–150 µm, depending on the smoothness of the extracted data. For a generally isotropic material, the group velocity of a shear wave can be alternatively determined from the spatiotemporal map by identifying the time points at which the wave peak arrived at individual azimuth positions (i.e. the time to peak), with least-square linear fitting applied to these points. For each imaged matrix, shear-wave measurements were performed repeatedly along directions crossing the matrix and equally separated by 45°. This was achieved by rotating the scanned sample relative to the imaging transducer. The SWEI measurement typically took 20–30 min for one sample. After the measurement, the sample was immediately placed into a new culture dish full of fresh culture medium, and placed back in the incubator.

### Shear rheology study

Rheological measurement of collagen matrices was conducted using a stress-controlled rheometer (AR2000ex, TA Instruments, DE) equipped with a 40 mm 4° steel cone at 37°C. A solvent trap was used throughout the measurement to prevent the gel from drying and the truncation gap was 103 µm. Gelation onset was determined by the time sweep measurement. The oscillating strain applied to the matrices was 1% and the frequency range was 0.1–30 Hz. The theoretical phase velocities as a function of angular frequency were derived using the measured storage and loss moduli at various frequencies as described previously (Bernal et al., 2013), namely:
(1)
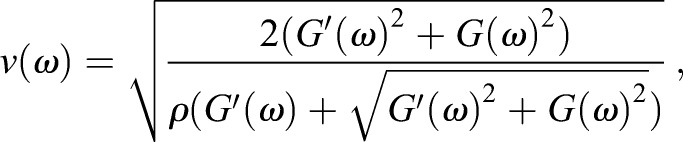
where *v*, *ρ*, *ω*, *G*′, and *G*″ represent the phase velocity, mass density of the material and angular frequency, as well as storage and loss modulus measured by the rheometer, respectively.

### Confocal imaging

The cell-populated collagen matrices were fixed with 4% paraformaldehyde, permeabilized using 0.01% Triton X-100, incubated with Hoechst 33342 (1:2000; H3570, Life Technologies, Waltham, MA), and examined with a confocal laser-scanning microscope (TCS SP5, Leica, Germany). Collagen fibrils were imaged using the emission at 488 nm.

### Drugs preparation

The BDM solution was purchased from Sigma-Aldrich (B0753) and added into the culture medium of the investigated samples to yield a final concentration of 40 mM. For the BAPN treatment, the cell-populated matrices were incubated daily with 350 μM BAPN prepared by dissolving 3-aminopropionitrile fumarate salt (A3134, Sigma-Aldrich) in culture medium. The BLEB solution was prepared by dissolving blebbistatin (B0560, Sigma-Aldrich) with DMSO into deionized water to yield a concentration of 30 μM.

### Statistical analysis

Results are all expressed as mean±s.d. Differences between data were evaluated by one-way ANOVA and a *P*<0.05 was considered to be significant.
